# Microshear bond strength of resin composite to Ti6A14V titanium alloy after different chemical and mechanical surface treatments

**DOI:** 10.1186/s12903-025-06614-x

**Published:** 2025-08-11

**Authors:** Reza Tayefeh Davalloo, Sanaz AziziGermi, Zeinab Moghaddami, Heshmatollah Ebrahimi-Najafabadi, Mehrsima Ghavami-Lahiji

**Affiliations:** 1 Department of Restorative Dentistry, Dental Sciences Research Center, School of Dentistry, Guilan University of Medical Sciences, Rasht, Iran; 2 Department of Medicinal Chemistry, School of Pharmacy, Guilan University of Medical Sciences, Rasht, Iran

**Keywords:** Shear strength, Titanium alloy (TiAl6V4), Dental implants, Acid etching, Dental, Hydrofluoric acid

## Abstract

**Background:**

This study aimed to compare the effects of different surface treatments including sandblasting, 9% hydrofluoric (HF) acid, 48% sulfuric acid (SA), and silica gel plus SA on micro-shear bond strength (μSBS) of resin composite to Ti6A14V titanium alloy.

**Methods:**

In this in vitro study, 60 Ti6A14V titanium alloy plates were randomly assigned to five experimental groups (*n* = 12) as follows: Group (1) untreated, Group (2) sandblasted (50 μm aluminum oxide particle), Group (3) acid etched in 9% HF for 60 s, Group (4) acid etched in 48% H_2_SO_4_ at 60 °C for 30 min, and Group (5) acid etched in 50% silica-sulfuric acid (SiO_2_–H_2_SO_4_) at 60 °C for 30 min. Profilometric examination and scanning electron microscopy (SEM) were performed. A universal adhesive resin was applied to the plates and light-cured. One tygon tubes was placed perpendicularly to each plate. The resin composite was then placed on the treated plates and light-cured for 40 s. μ-SBS and failure mode were analyzed after 2000 thermal cycles (5 to 55 °C). Data were analyzed by ANOVA, Tukey, and Chi-square tests at the significance level of 0.05.

**Results:**

The μSBS was significantly different among the groups (*P* = 0.032). The mean µSBS of the control group was significantly lower than that of sandblasting (*P* < 0.001), SA etching (*P* = 0.009) and silica-SA (*P* = 0.003) groups. The Ra of sandblasting (*P* = 0.021) and silica-SA (*P* = 0.004) groups was significantly higher than the control group. Also, the Ra of HF acid group was significantly lower than that of sandblasting (*P* = 0.035) and silica-SA (*P* = 0.007) groups. The Rz of sandblasting group was significantly higher than all other groups (*P* < 0.001 for all).

**Conclusion:**

Within the study limitations, surface treatment by sandblasting resulted in the highest surface roughness and μSBS of Ti6A14V alloy-resin composite followed by silica gel-SA, SA etching, and HF.

**Supplementary Information:**

The online version contains supplementary material available at 10.1186/s12903-025-06614-x.

## Introduction

Single implant crowns were introduced in 1996 as the treatment of choice for replacement of the lost teeth [1]. Since dental implants are in direct contact with the tissue and are exposed to the physical and chemical oral bio-environment, their biocompatibility is a necessity [2]. Titanium (Ti) and its alloys have unique biological and mechanical properties, making them an ideal material for the fabrication of dental implants and abutments. Other materials such as zirconia have higher biological complications than titanium, and it has been confirmed that bone and soft tissue loss would be minimum in case of using titanium, compared with any other material. Due to high strength and optimal biocompatibility, titanium and its alloys such as titanium-6% aluminum-4% vanadium (Ti6Al4V) are currently the material of choice for dental implants [3, 4].

A successful dental implant treatment requires not only a properly inserted fixture, but also a suitable prosthetic restoration and supra-structure, and a correct dental implant-abutment assembly. A thorough understanding of the relationship of dental implant and the adjacent soft tissue is also important in achieving optimal esthetics [5–7].

In clinical practice, temporary restorations are sometimes directly applied to standard abutments (e.g., modifying provisional crowns with resin-based materials on standard abutments). This approach allows clinicians to evaluate functional and aesthetic outcomes while enabling controlled occlusal adjustments prior to definitive prosthesis delivery. Such temporary phases are particularly critical in cases involving short abutments, traumatic occlusion, or complex full-mouth rehabilitations requiring interim stabilization [8–10].

Thus, resin bonding to Ti6Al4V abutments may be essential in some key scenarios: (1) cementation protocols requiring enhanced initial retention for stabilization [11], particularly in full-mouth rehabilitations where resin cements must withstand prolonged occlusal forces during transitional phases; (2) Interim prosthetic phases during extensive occlusal rehabilitations, where interceptive prostheses on standard abutments require precise occlusal stabilization prior to definitive restoration [12]; (3) Screw-retained prostheses with Ti-Base connections bonded to zirconia crowns, where inadequate bonding to resin cements risks crown displacement under functional loads [13]. Additionally, gingival contour modifications during direct relining of provisional restorations demand durable resin-abutment bonds to maintain retention while achieving optimal emergence profiles [4, 6, 14, 15].

Although resins are suitable for the abovementioned purposes, they cannot chemically bond to Ti6Al4V alloy used in implant abutments, making this process challenging [1, 4, 16, 17]. To overcome this problem, the abutment manufacturers suggested macro-mechanical retention; although practical, this technique did not have the unique properties of the etching technique, which is commonly used for composite restorations. Another suggested strategy to increase the contact surface area was to perform titanium etching by using acidic solutions. Accordingly, titanium-composite bonding may be used for esthetic purposes or making the necessary corrections in the abutments. Considering the high biocompatibility and very high corrosion resistance of titanium, this technique may efficiently prepare the surface of titanium alloy for bonding to other dental materials [6, 9, 18].

Different surface treatments are available for this purpose, which may be divided into mechanical, chemical, and electrochemical methods [19]. To date, several methods have been proposed to create a strong bond between the metal substrate and resin materials, such as air abrasion with aluminum oxide particles, or a combination of sandblasting and hydrofluoric (HF) acid etching, application of materials containing functional monomers such as 10-MDP, creation of a retentive groove in the abutment body, application of alloy primer, tribochemical silica coating, and laser irradiation [11, 20]. Nonetheless, the efficacy of titanium etching for this purpose is still a matter of question and requires further investigations [18, 21, 22].

On the other hand, application of strong acids for titanium surface etching is hazardous, and some safety measures need to be taken. To minimize the risks of liquid acids and evaporation of acidic gases, a reagent such as silica may be used to minimize the side effects of acids and ensure the safety of acid application while preserving the structure and efficacy of acids in the clinical setting [22, 23].

To the best of the authors’ knowledge, no previous study has compared the efficacy of different titanium etching protocols under controlled conditions, and the available relevant studies only assessed the efficacy of one single etching protocol independently [3, 7, 11, 18, 20, 24, 25].

Considering all the above, this study aimed to compare the effects of different surface treatments including sandblasting, 9% HF acid, 48% sulfuric acid (SA), and silica gel plus SA (as a novel experimental etchant gel to control the consistency of SA and enhance its application) on micro-shear bond strength (μSBS) of resin composite to Ti6A14V titanium alloy. Null Hypotheses were that: There are no significant morphological changes (e.g., oxide layer removal, surface topography alterations) and no significant differences in surface roughness of titanium surfaces treated with different surface modification technique: There is no significant difference in μSBS of resin composite to Ti6Al4V titanium alloy among the different surface treatments.

## Materials and methods

This in vitro, experimental study was conducted on 58 Ti6A14V titanium alloy plates.

### Sample size

The minimum sample size was calculated to be 9 for each group assuming alpha = 0.05, study power of 0.95, and standard deviations of μSBS to be 0.931 for the control group and 1.419 for the test group according to a previous study [18], using the feature for comparison of the two means and adjusting it for the comparison of 5 groups. To increase accuracy, 12 specimens were considered for each group.

### Specimen preparation

A total of 60 titanium plates measuring 10 × 10 × 2 mm were fabricated from Ti6Al4V alloy (with a chemical composition corresponding to grade 5). According to a study by Alberti et al., [18] this alloy matched the titanium alloy used in dentistry for abutment fabrication in terms of composition and percentage of chemical elements. The surface of the specimens was polished with 180-, 320-, 400-, 600-, and 1200-grit silicon carbide abrasive papers under water coolant. They were then cleaned in an ultrasonic bath (Elmasonic, Elma S, NJ, USA) containing acetone for 10 min and dried calculated and reported.

### Interventions

The specimens were randomly assigned to 5 groups (*N* = 12) and underwent the following surface treatments by one operator:

- Group 1 (control): This group did not receive any surface treatment.
- Group 2: The specimens in this group underwent air abrasion by sandblasting with 50 µm aluminum oxide particles with 60 Psi pressure for 10 s through a nozzle held at 10 mm distance from the specimen surface at 45-degree angle [26]. The specimens were then rinsed under running water and cleaned in an ultrasonic bath containing acetone for 10 min. They were then rinsed with water for 2 min and air dried.
- Group 3: The specimens underwent etching with 9% HF acid gel (Porcelain Etch,Ultradent™, USA) for 60 s [18]. They were then rinsed under running water and placed in an ultrasonic bath containing acetone for 10 min. Finally, they were rinsed with water for 2 min and air dried.
- Group 4: The specimens underwent etching with 48% SA (H_2_SO_4_, Merck, Germany) and etched for 30 min at 60 °C in a water vapor chamber [18, 23]. To prepare 10 mL of 48% SA, 2.67 mL of 98% acid was reached to 10 mL volume with distilled water. The specimens were then carefully rinsed with water and placed in an ultrasonic bath containing acetone for 10 min. Finally, the specimens were rinsed with water for 2 min and air-dried.
- Group 5: The specimens underwent etching by using an experimental etchant composed of silica gel and 50% SA, and etched for 30 min at 60 °C in a water vapor chamber. To synthesize the experimental etchant, 20 g of silica gel 60 (Merck, Germany) was added to 25 mL of 74% SA solution and placed on a rotary evaporator for 5 h. The obtained paste-like deposits were used for etching. The specimens were then carefully rinsed with water and placed in an ultrasonic bath containing acetone for 10 min. Finally, the specimens were rinsed with water for 2 min and air-dried.

### Surface roughness

The surface roughness was measured by measuring the Ra and Rz values of each specimen by a contact profilometer (Hommelwerke, Germany) with 5 mm measurement length, speed of 0.6 mm/second and accuracy of 0.01 µm at three points, and the mean of the three values was.

### Surface topography

The surface topography of one specimen from each group was sputter-coated with gold and assessed under a scanning electron microscope (SEM; TESCAN, VEGA, Czech Republic) at × 10,000 magnifications.

### Measurement of μSBS

A universal adhesive containing 10-MDP active monomer (Ambar Universal Bond; FGM dental group, Brazil) was first applied on the surface of plates by a microbrush and gently air dried to standardize the thickness and decrease the solvent. Table 1 presents the composition of the adhesive and resin composite used in this study. The adhesive was light-cured with a LED curing unit (Bluedent, LED Smart, Bulgaria) with a light intensity of 1000 mW/cm^2^ for 20 s. The intensity of the output light was calibrated by a radiometer (LM-100; DigiRate). Next, a nano-hybrid restorative resin composite (Opalis; FGM Joinville, SC, Brazil) was applied into a plastic tube with an internal diameter of 1 mm and 2 mm height in two 1-mm thick increments (to reach 2 mm thickness) on the specimen surface. The resin composite was applied in a single increment of 2 mm to each specimen. Composite placement was performed under 2 × magnification using a dental loupe to ensure precise positioning and minimize voids or uneven surfaces. The plastic tubes were then gently cut by a sharp scalpel, and the composite cylinder was once again light-cured for 40 s from two opposite directions (each for 20 s). Specimens were inspected to discard any with flaws, air bubbles, or gaps at the interface. The specimens were then immersed in distilled water at 37 °C for 24 h. The specimens subsequently underwent thermocycling (Nemo Mecatronica, Mashhad, Iran) for 2000 cycles in water baths between 5–55°C with a dwell time of 20 s and a transfer time of 20 s.

**Table 1:** Composition of the adhesive and composite resin used in the present study

Material type	Brand	Composition	Manufacturer
Nanohybrid restorative composite	Opallis	Bis-GMA (Bis-Phenol A di-Glycidyl Methacrylate); BisEMA (Bis-Phenol A di-Glycidyl Ethoxylated methacrylate); TEGDMA (Triethylene glycol dimethacrylate); UDMA (Urethane dimethacrylate); camphorquinone, co-initiator silane. Inactive ingredient: silanized barium-aluminum silicate glass, pigments, and silica.	**FGM Joinville, SC, Brazil**
Univ ersal adhesive system	Ambar APS	Active ingredients: MDP (10Methacryloyloxidecyl dihydrogen phosphate), methacrylic monomers, photo initiators, co-initiators, and stabilizer. Inactive ingredients: inert filler (silica nanoparticles), and vehicle (ethanol).	**FGM Joinville, SC, Brazil**

Next, the specimens were fixed to the jig of a universal testing machine (STM-20; Santam Series, Iran) with cyanoacrylate glue, and subjected to μSBS by using a 6-kg load cell. A stainless-steel orthodontic wire with 0.2 mm diameter was looped around the composite cylinder, and gently held against the composite-titanium interface. Load was applied through this wire loop as close to the composite-titanium interface as possible at a crosshead speed of 0.5 mm/minute. Load at the time of debonding in Newtons (N) was recorded and divided by the bonding cross-sectional area in square millimeters (mm^2^) to calculate the μSBS in megapascals (MPa) [27, 28].

### Mode of failure

The mode of failure of the broken specimens was determined under a stereomicroscope (EchoLab, DEVCO s.r.l, Italy) at × 40 magnification. The mode of failure was categorized as follows:

(I) Adhesive failure at the composite-titanium interface
(II) Cohesive failure in the resin composite
(III) Mixed failure (a combination of adhesive and cohesive failures)

### Statistical analysis

Data were analyzed using SPSS version 23 (SPSS Inc., IL, USA). The Shapiro–Wilk test was used to analyze the normality of data distribution while the Levene’s test was applied to assess the homogeneity of the variances. Accordingly, comparisons were made with ANOVA, Tukey’s test (for pairwise comparisons), and the Chi-square test at 0.05 level of significance.

## Results

### Surface roughness

Table 2 presents the mean Ra and Rz values of the groups. The highest surface roughness was noted in silica gel-SA group and the lowest in HF acid group. ANOVA revealed a significant difference in surface roughness among the groups (*P* = 0.003). Pairwise comparisons revealed that the Ra of sandblasting (*P* = 0.021) and silica-SA (*P* = 0.004) groups was significantly higher than the control group. Also, the Ra of the HF acid group was significantly lower than that of the sandblasting (*P* = 0.035) and silica-SA (*P* = 0.007) groups. The Rz value of the sandblasting group was significantly higher than all other groups (*P* < 0.001 for all). No other significant differences were found (*P* > 0.05).

**Table 2:** Mean Ra and Rz values of the groups

Group	Ra (μm)	Rz (μm)
	Sd ± Mean	SD ± Mean
Control	0.06 ± 0.61	49/0 ± 68/3
Sandblast	0.11± 0.77	6.42 ± 0.49
HF acid	0.11 ± 0.62	3.99 ± 0.44
SA	0.04 ± 0.68	4.11 ± 0.32
Silica-SA	0.07 ± 0.80	4.44 ± 0.28

### SEM results

**Fig. 1:**
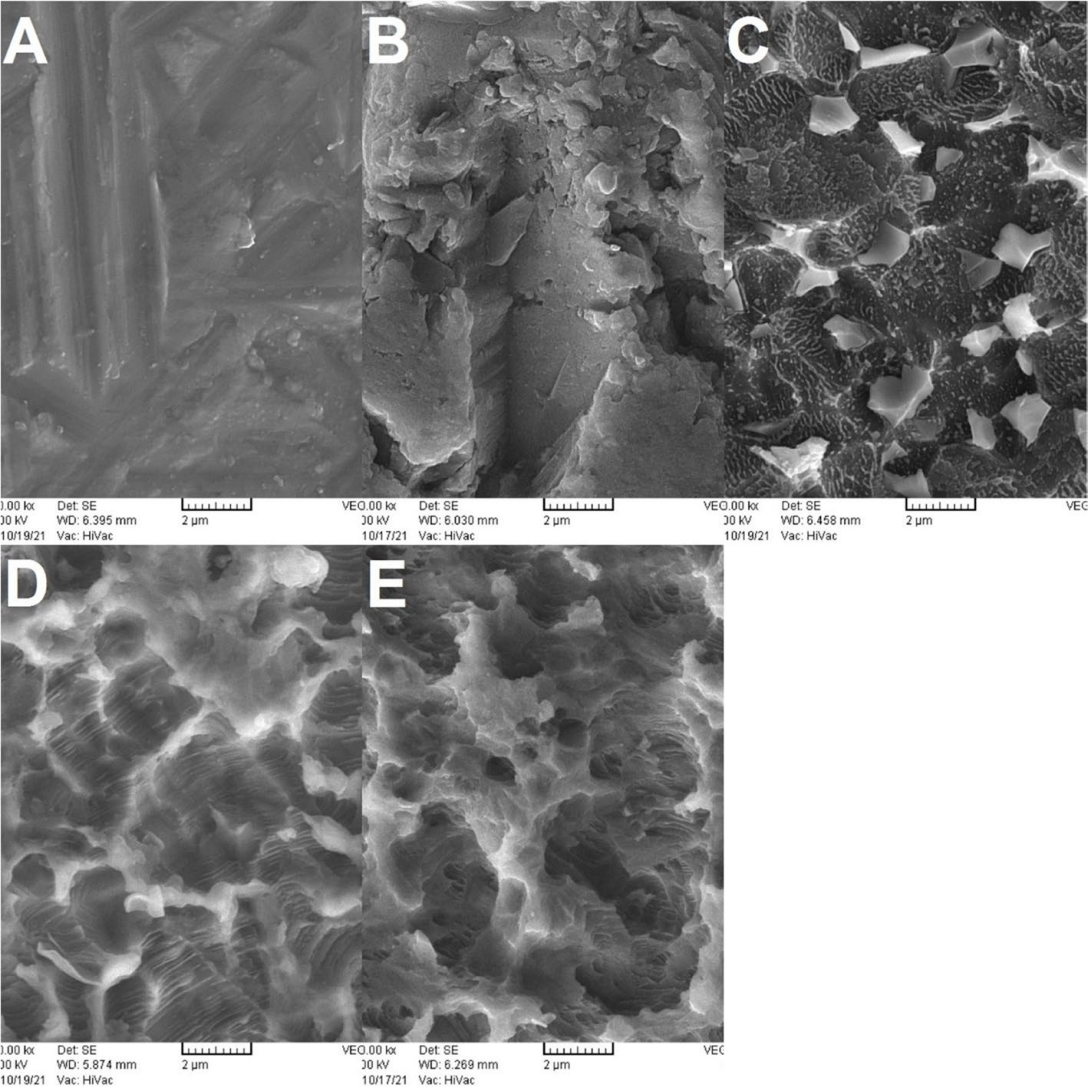
SEM micrographs of the surface of specimens at × 10,000 magnification: (**A**) control group; (**B**) sandblasting group showing nanoholes, microporosities, and oxide layer removal; (**C**) HF acid group with residual oxide layer and limited porosity; (**D**) SA group and (**E**) silica-SA group demonstrating uniform phase-selective etching and increased porosity vs. control, contrasting with sandblasting’s mechanical abrasion patterns

Figure 1 show the SEM micrographs of the surface of the specimens.

### µSBS

Table 3 presents the mean µSBS of the groups. As shown, the highest µSBS was recorded in the sandblasting group, and the lowest in the HF acid etching group. ANOVA revealed a significant difference in µSBS among the groups (*P* = 0.021). Thus, pairwise comparisons were performed by the Tukey’s post-hoc test, which revealed that the mean µSBS of the control group was significantly lower than that of the sandblasting (*P* < 0.001), SA etching (*P* = 0.009) and silica-SA (*P* = 0.003) groups. No other significant differences were found (*P* > 0.05). The regression model showed that type of etchant was a predictive factor for µSBS (*P* < 0.001, *r* = 0.433).

**Table 3:** Mean µSBS (MPa) of the groups

Group	Mean	Std. deviation
Control	8.54	2.62
Sandblast	17.43	5.29
HF acid	14.12	4.42
SA	15.36	2.49
Silica-SA	15.99	5.38

It was found that Ra (*P* = 0.032, *r* = 0.332) and Rz (*P* = 0.022, *r* = 0.352) values had a significant correlation with µSBS.

### Mode of failure

There were 30 (51.7%) adhesive failures, 2 (3.4%) cohesive failures, and 26 (44.8%) mixed failures (Table 4). The Chi-square test showed no significant difference among the groups in the mode of failure (*P* = 0.287).

**Table 4:** Frequency of different modes of failure in the study groups

Group	Frequency		
	Adhesive	Cohesive	Mixed
Control	11	0	1
Sandblast	4	0	8
HF acid	6	0	6
SA	5	1	6
Silica-SA	6	1	5

## Discussion

The superstructure of dental implants is a determining factor in providing esthetic, function, and longevity. The success of customized superstructure as an unavoidable process in some clinical situations relays heavily on the composite-to-titanium bonding strength. This study aimed to characterize the surface roughness and topography of the treated Ti6Al4V alloy using three different etchants and sandblast and evaluate the micro-shear bond strength (μ-SBS) of a bonded resin composite to Ti6Al4V. Silica gel plus SA was used as a novel experimental etchant gel to control the consistency of SA and enhance its application. Our findings indicated that the hypotheses were rejected, as surface treatments created different surface roughness, μSBS, and morphological changes among groups.

The results showed the highest µSBS in the sandblasting group, followed by silica-SA, and SA group. The µSBS in the sandblasting, silica-SA and SA groups was significantly higher than the control group while HF acid group had no significant difference with the control group in µSBS. Venkat et al. [11] showed that sandblasting, HF acid etching, and laser irradiation all yielded an acceptable µSBS to titanium, which was significantly higher than the µSBS in the control group. They combined 1% HF acid with 30% nitric acid and reported that it was a strong etchant for the titanium alloy, yielding a µSBS almost comparable to that of sandblasting in bonding of composite to titanium alloy. Moeeduddin et al. [29] concluded that concentration of etchant had no significant effect on the bond strength while the effect of etching time was significant. They found no significant difference in bond strength provided by etching with 5% and 9% HF acid. Parchanska-Kowalik et al. [30] evaluated the efficacy of etching with HF acid alone, and in combination with hydrogen peroxide, nitric acid and nitric acid plus glycerin for etching of CpTi1 titanium. They demonstrated that all tested products were effective for etching of titanium and yielded significantly superior results compared with the control group. Nonetheless, the mean µSBS of the HF acid group in the present study was slightly, but not significantly, higher than the control group. This difference in the results may be due to different etching times as an influential parameter on bond strength, or using different types of titanium alloys in different studies. According to Chauhan et al., [31] the Ti6Al4V alloy has a totally different behavior, topography, and surface roughness compared with the CpTi alloy upon acid exposure and after acid etching (with HF, HCl, or H_2_SO_4_). It is clear that surface topography and surface roughness directly determine the level of micromechanical retention and bond strength [31]. Another study on tensile bond strength of cast metal copings cemented to commercial Cp-Ti analogs revealed the highest bond strength in sandblasting plus acid etching group while sandblasting alone yielded the lowest bond strength. Acid etching alone resulted in a moderate bond strength. They used 48% SA at 60 °C for 1 h and then cleaned the specimens in an ultrasonic bath containing NaHCO_3_ [7].

The current results regarding significant enhancement of bond strength by surface treatment with sandblasting and SA etching were in agreement with the available literature in this regard [23, 32, 33]. Nonetheless, it should be noted that previous studies evaluated Cp-Ti alloy while the current study was conducted on Ti6A14V alloy.

In the present study, combined use of SA with silica gel (for easier handling and superior safety) yielded significantly higher µSBS than the control group but had no superiority over SA alone. Thus, its application is suggested for titanium etching due to its comparable efficacy but higher level of safety and easier handling compared to SA etching alone.

Universal adhesive contains was used in this study. Studies demonstrate that 10-MDP primers significantly enhance resin bond strength to titanium compared to other primers (e.g., 4-MET) [34].

Assessment of the surface roughness of titanium surfaces in the present study revealed the highest Rz value in the sandblasting group and comparable Ra values in the sandblasting and silica-SA groups, which were in accordance with the results of µSBS testing. The surface roughness of the sandblasting and silica-SA groups was significantly higher than that of the control group, and a significant correlation was found between surface roughness and µSBS, which was expected. HF acid etching could not significantly increase the surface roughness compared with the control group. Consistent with the present results, Zareidoost et al. [35] demonstrated that application of HF acid alone for etching of titanium surfaces could not significantly change the texture of the titanium surface compared to the control group while, a combination of HF acid with HCl and H_3_PO_4_ was significantly effective in creating a rough titanium surface. Also, Rosales-Leal et al. [36] indicated that HF acid etching created the smallest changes in the titanium surface while sandblasting prior to HF acid etching resulted in the greatest surface alterations. Sandblasting alone was more effective than acid etching and less effective than the combination of sandblasting and HF acid etching in their study. Ban et al. [23] revealed that SA etching caused significant roughening of the titanium surface. They stated that acid concentration, temperature, and etching time are the key parameters determining the efficacy of acid in roughening of titanium surfaces. The results of the abovementioned studies were almost in line with the present findings with the difference that the authors of the present study aimed to find a safe, efficient, and easy method for clinical use with no need for advanced equipment. Thus, the lowest concentration of acids for the shortest time were used in the present study for higher level of safety. Also, silica gel was used in combination with SA to enhance its handling and safety, which showed optimal results in terms of bond strength and surface roughness. Considering the reportedly positive efficacy of silica-coating [20], optimal results can be due to the chemical reaction of the silicated surface (even in presence of small residual silica on the etched metal surface) in combination with chemical surface etching. Also, the effects of time and concentration of etchant on surface roughness and bond strength may be due to degradation of the superficial oxide layer of titanium following exposure of the metal surface to high temperature, or high concentration of acid, or prolonged exposure. Obviously, presence of a thick superficial oxide layer on the titanium surface has an inverse correlation with bond strength of composite to titanium. Also, Ezoji et al. [37] evaluated the effect of Nano Met Etch gel in comparison with sandblasting and found that it had a comparable or even superior effect than sandblasting on nickel–chromium alloy while it was easier to use than sandblasting. Nonetheless, sandblasting was superior to acid etching for metals with high silver content.

Comparison of SEM micrographs revealed significant differences in surface roughness and surface porosities of the experimental groups compared with the control group in all three magnifications. All surface-treated specimens had surface irregularities and porosities due to exposure of different phases of Ti–Al-V. SEM micrographs of the sandblasted specimens revealed formation of nanoholes along with almost complete removal of the superficial oxide layer of titanium and formation of numerous micro-porosities. The surface porosity in the HF acid group was much lower than the sandblast group and other experimental groups, and a residual surface oxide layer was also observed in this group, which confirmed the low surface roughness and bond strength of this group compared to other groups. Although this group had slightly higher surface porosities than the control group, they were obviously not sufficient to yield optimal surface roughness and bond strength (Fig. 1). Peláez-Abellán et al. [38] showed that increasing the etching time of Cp-Ti with 2% HF acid from 30 to 180 s increased the dissolution of the oxide layer, and its complete dissolution occurred after 180 s. Although a higher concentration of HF acid was used in the present study, 60 s of etching time was obviously not sufficient for complete elimination of the oxide layer. Nonetheless, it should be noted that Ti6Al4V alloy was used in the present study, which is a crystalline material with α + β phases, and since the speed and magnitude of etching vary for Ti, Al, and V elements, differences in surface roughness and topography compared with Cp-Ti are expected.

In the SA and silica-SA groups, the etching pattern and exposed structure of different titanium phases were highly similar, but the porosities were much higher compared with the control group (Fig. 1). Nonetheless, the size of exposed particles and surface morphology were different from those in the sandblasting group, and a more uniform surface morphology and a more regular etching pattern were seen in these two groups compared with the sandblasted group, which may be explained by the mechanical nature of sandblasting.

Regarding the mode of failure, adhesive and mixed failures were equally dominant, which was expected considering the obtained bond strength values, and no significant difference was noted in the mode of failure among the groups.

This study had some limitations. Considering the cylindrical shape of titanium abutments and their high hardness, their sectioning for μSBS testing was difficult and costly; thus, titanium plates were used instead. Also, the minimum concentration and shortest time of exposure of acids were adopted for the present study to simplify the process and increase the level of safety. Thus, comparison of different acid concentrations and exposure times could not be performed.

Future studies may evaluate different concentrations and longer etching times, and assess the efficacy of combined surface treatments to achieve a higher bond strength. Also, alloy primers or metal primers containing 4-META may be used in further investigations to assess their efficacy.

In addition to the current focus on titanium alloys, future research directions could also explore the application of surface treatments to other materials commonly used in longer-term interim restorations. For instance, CAD/CAM composite or PEEK blocks are often utilized when a longer interim period is anticipated. In such scenarios, the bonding of composite to these materials becomes a significant concern. Therefore, investigating the effectiveness of surface treatments for improving the adhesion of composite to CAD/CAM composite or PEEK materials would be a valuable next step.

## Conclusion

Within the study limitations, the results showed that surface treatment of Ti6A14V titanium alloy with sandblasting, silica gel-SA and SA etching yielded the highest surface roughness and μSBS of titanium- resin composite. Surface modification of Ti6Al4V with experimental SiO_2_–H_2_SO_4_ can be a promising method for composite-to-titanium bonding purposes.

## Supplementary Information

Supplementary Material 1.

Supplementary Material 2.

Supplementary Material 3.

Supplementary Material 4.

Supplementary Material 5.

## Data Availability

No datasets were generated or analysed during the current study.

## References

[1] Hall JA, Payne AG, Purton DG, Torr B, Duncan WJ, De Silva RK. Immediately restored, single-tapered implants in the anterior maxilla: prosthodontic and aesthetic outcomes after 1 year. Clin Implant Dent Rel Res. 2007;9(1):34–45. 10.1111/j.1708-8208.2007.00029.x 17362495

[2] Oshida Y. Bioscience and Bioengineering of Titanium Materials. Amsterdam: Elsevier; 2010.

[3] Kemarly K, Arnason S, Parke A, Lien W, Vandewalle K. Effect of various surface treatments on Ti-base coping retention. Oper Dent. 2020;45(4):426–34. 32053453 10.2341/19-155-LR

[4] Beretta M, Poli PP, Pieriboni S, Tansella S, Manfredini M, Cicciù M, et al. Peri-implant soft tissue conditioning by means of customized healing abutment: a randomized controlled clinical trial. Materials. 2019;12(18): 3041. 31546800 10.3390/ma12183041 PMC6766291

[5] Potashnick SR. Soft tissue modeling for the esthetic single-tooth implant restoration. J Esthet Restor. 1998;10(3):121–31. 10.1111/j.1708-8240.1998.tb00348.x 9759029

[6] Wadhwani CP, Schoenbaum T, King KE, Chung K-H. Techniques to optimize color esthetics, bonding, and peri-implant tissue health with titanium implant abutments. Compend Contin Educ Dent. 2018;39(2):110–9. 29388785

[7] Ajay R, Rakshagan V, Kamatchi M, SelvaBalaji A, Sivakumar JSK, Kumar MS. Effect of implant abutment acid etching on the retention of crowns luted with different cements: an: in vitro: comparative evaluation. J Pharm Bioallied Sci. 2019;11(Suppl 2):S360–4. 31198369 10.4103/JPBS.JPBS_35_19 PMC6555309

[8] Shrivastav M. Effect of surface treatments on the retention of implant-supported cement-retained bridge with short abutments: an: in vitro: comparative evaluation. J Indian Prosthodont Soc. 2018;18(2):154–60. 29692569 10.4103/jips.jips_251_17 PMC5903179

[9] Wadhwani C, Chung K-H. In-office technique for selectively etching titanium abutments to improve bonding for interim implant prostheses. J Prosthet Dent. 2016;115(3):271–3. 26553255 10.1016/j.prosdent.2015.07.018

[10] Dua B, Gupta R, Bhargava A, Mittal N. Advancements in surface treatment techniques for dental implants: enhancing osseointegration and clinical outcomes. J Chem Health Risks. 2024;14:1927–41.

[11] Venkat G, Krishnan M, Srinivasan S, Balasubramanian M. Evaluation of bond strength between grooved titanium alloy implant abutments and provisional veneering materials after surface treatment of the abutments: an: in vitro: study. Contemp Clin Dent. 2017;8(3):395–9. 29042724 10.4103/ccd.ccd_118_17 PMC5643996

[12] Kaur JB, Singh K, Mathew RC, JH J. Complete occlusal rehabilitation of a grossly decayed natural dentition using multiple prosthodontic options. Am J Med Case Rep. 2023;11(3):43–8.

[13] Zahoui A, Bergamo ET, Marun MM, Silva KP, Coelho PG, Bonfante EA. Cementation protocol for bonding zirconia crowns to titanium base CAD/CAM abutments. Int J Prosthodont. 2020;33(5):527–35. 32956434 10.11607/ijp.6696

[14] Ilie N, Hilton T, Heintze S, Hickel R, Watts D, Silikas N, et al. Academy of dental materials guidance—resin composites: part I—mechanical properties. Dent Mater. 2017;33(8):880–94. 28577893 10.1016/j.dental.2017.04.013

[15] Lee EA. Transitional custom abutments: optimizing aesthetic treatment in implant-supported restorations. Pract Periodontics Aesthet Dent. 1999;11(9):1027–34. 10853587

[16] Block M, Finger I, Castellon P, Lirettle D. Single tooth immediate provisional restoration of dental implants: technique and early results. J Oral Maxillofac Surg. 2004;62(9):1131–8. 15346366 10.1016/j.joms.2004.05.115

[17] Henriksson K, Jemt T. Measurements of soft tissue volume in association with single-implant restorations: a 1-year comparative study after abutment connection surgery. Clin Implant Dent Relat Res. 2004;6(4):181–9. 15841578 10.1111/j.1708-8208.2004.tb00034.x

[18] Alberti CJ, Saito E, Freitas FEd, Reis DAP, Machado JPB, Reis AGd. Effect of etching temperature on surface properties of Ti6Al4V alloy for use in biomedical applications. Mater Res. 2019;22(suppl. 1): e20180782.

[19] Salgado L, Zayas T, Rodríguez CB, Soriano-Moro G, Lara VH. Titanium surface treatments and their effects on the roughness factor. Int J Electrochem Sci. 2024;19(8): 100697.

[20] Elsharkawy S, Shakal M, Elshahawy W. Effect of various surface treatments of implant abutment and metal cope fitting surface on their bond strength to provisional resin cement. Tanta Dent J. 2015;12(4):235–40.

[21] Murray AK, Attrill DC, Dickinson MR. Qualitative assessment of surface topography of XeCl laser etched Ni–Cr alloy. Dent Mater. 2005;21(9):837–45. 16087005 10.1016/j.dental.2005.01.003

[22] Hsiao VK, Shih M-H, Wu H-C, Wu T-I. Comparative study of surface modification techniques for enhancing biocompatibility of Ti-6Al-4V alloy in dental implants. Appl Sci. 2024;14(23):10904.

[23] Ban S, Iwaya Y, Kono H, Sato H. Surface modification of titanium by etching in concentrated sulfuric acid. Dent Mater. 2006;22(12):1115–20. 16375960 10.1016/j.dental.2005.09.007

[24] Chaijareenont P, Prakhamsai S, Silthampitag P, Takahashi H, Arksornnukit M. Effects of different sulfuric acid etching concentrations on PEEK surface bonding to resin composite. Dent Mater J. 2018;37(3):385–92. 29375092 10.4012/dmj.2017-141

[25] Turker N, Özarslan MM, Buyukkaplan US, Başar EK. Effect of Different Surface Treatments Applied to Short Zirconia and Titanium Abutments. Int J Oral Max Implants. 2020;35(5):948–54. 10.11607/jomi.8224. 10.11607/jomi.8224 32991645

[26] Banerjee A, Thompson I, Watson T. Minimally invasive caries removal using bio-active glass air-abrasion. J Dent. 2011;39(1):2–7. 20888883 10.1016/j.jdent.2010.09.004

[27] Cengiz-Yanardag E, Karakaya I. The effect of resveratrol application on the micro-shear bond strength of adhesive to bleached enamel. Sci Rep. 2024;14(1):24201. 39406800 10.1038/s41598-024-75024-w PMC11480448

[28] Rezaee M, Valian A, Tavakoli A, Nikaein M. Effect of internal bleaching on the microshear bond strength of composite resin to dentin in recently restored teeth. J Iran Med Council. 2023;6(4):712–8.

[29] Moeeduddin M, Nathanson D, Fan Y. Effect of firing cycle and etching protocols on tensile bond strength of composite cement to zirconium-incorporated lithium-silicate glass ceramic. J Adhes Dent. 2020;22:625–33. 33491406 10.3290/j.jad.a45518

[30] Parchańska-Kowalik M, Wołowiec-Korecka E, Klimek L. Effect of chemical surface treatment of titanium on its bond with dental ceramics. J Prosthet Dent. 2018;120(3):470–5. 29627218 10.1016/j.prosdent.2017.11.025

[31] Chauhan P, Koul V, Bhatnagar N. Critical role of etching parameters in the evolution of nano micro SLA surface on the Ti6Al4V alloy dental implants. Materials. 2021;14(21): 6344. 34771869 10.3390/ma14216344 PMC8585160

[32] Sproesser O, Schmidlin PR, Uhrenbacher J, Roos M, Gernet W, Stawarczyk B. Effect of sulfuric acid etching of polyetheretherketone on the shear bond strength to resin cements. J Adhes Dent. 2014;16(5):465–72. 10.3290/j.jad.a32806. 10.3290/j.jad.a32806 25264546

[33] Egoshi T, Taira Y, Soeno K, Sawase T. Effects of sandblasting, H_2_SO_4_/HCl etching, and phosphate primer application on bond strength of veneering resin composite to commercially pure titanium grade 4. Dent Mater J. 2013;32(2):219–27. 23538756 10.4012/dmj.2012-261

[34] Tsuchimoto Y, Yoshida Y, Mine A, Nakamura M, Nishiyama N, Van Meerbeek B, et al. Effect of 4-MET-and 10-MDP-based primers on resin bonding to titanium. Dent Mater J. 2006;25(1):120–4. 16706306 10.4012/dmj.25.120

[35] Zareidoost A, Yousefpour M, Ghaseme B, Amanzadeh A. The relationship of surface roughness and cell response of chemical surface modification of titanium. J Mater Sci Mater Med. 2012;23:1479–88. 22460230 10.1007/s10856-012-4611-9 PMC3368253

[36] Rosales-Leal JI, Rodríguez-Valverde MA, Mazzaglia G, Ramón-Torregrosa PJ, Díaz-Rodríguez L, García-Martínez O, et al. Effect of roughness, wettability and morphology of engineered titanium surfaces on osteoblast-like cell adhesion. Colloids Surf A: Physicochem Eng Asp. 2010;365(1–3):222–9.

[37] Ezoji F, Tabari K, Ansari ZJ, Torabzadeh H. Shear bond strength of a resin cement to different alloys subjected to various surface treatments. J Dent (Tehran). 2016;13(1):29. 27536326 PMC4983563

[38] Peláez-Abellán E, Duarte LT, Biaggio SR, Rocha-Filho RC, Bocchi N. Modification of the titanium oxide morphology and composition by a combined chemical-electrochemical treatment on cp Ti. Materials Research. 2012;15:159–65.

