# Correction: Microshear bond strength of resin composite to Ti6Al4V titanium alloy after different chemical and mechanical surface treatments

**DOI:** 10.1186/s12903-026-07661-8

**Published:** 2026-02-20

**Authors:** Reza Tayefeh Davalloo, Sanaz AziziGermi, Zeinab Moghaddami, Heshmatollah Ebrahimi-Najafabadi, Mehrsima Ghavami-Lahiji

**Affiliations:** 1 Department of Restorative Dentistry, Dental Sciences Research Center, School of Dentistry, Guilan University of Medical Sciences, Rasht, Iran; 2 Department of Medicinal Chemistry, School of Pharmacy, Guilan University of Medical Sciences, Rasht, Iran

**Correction: BMC Oral Health 25, 1314 (2025)**

**https://doi.org/10.1186/s12903-025-06614-x**

In Table 2 of this article [1], the order of Means and SDs and the control group value 49/0 ± 68/3 in column headed Rz (µm) was incorrect and should have been 3.68 ± 0.49.

In addition, the term "Ti6A14V" should be replaced with "Ti6Al4V" throughout the text, figure captions, and table headings.

Incorrect Table 2:

**Table 2:** Mean Ra and Rz values of the groups

Group	Ra (μm)Sd ± Mean	Rz (μm)SD ± Mean
Control	0.06 ± 0.61	49/0 ± 68/3
Sandblast	0.11± 0.77	6.42 ± 0.49
HF acid	0.11 ± 0.62	3.99 ± 0.44
SA	0.04 ± 0.68	4.11 ± 0.32
Silica-SA	0.07 ± 0.80	4.44 ± 0.28

Correct Table 2:

**Table 2:** Mean Ra and Rz values of the groups

Group	Ra (μm)Sd ± Mean	Rz (μm)SD ± Mean
Control	0.61 ± 0.06	3.68 ± 0.49
Sandblast	0.77 ± 0.11	6.42 ± 0.49
HF acid	0.62 ± 0.11	3.99 ± 0.44
SA	0.68 ± 0.04	4.11 ± 0.32
Silica-SA	0.80±0.07	4.44 ± 0.28
