# Supplementary figures and images for: Microshear bond strength of resin composite to Ti6A14V titanium alloy after different chemical and mechanical surface treatments
Source: BMC Oral Health. 2025 Aug 11;25:1314. doi: 10.1186/s12903-025-06614-x (PMC12337520; doi:10.1186/s12903-025-06614-x)

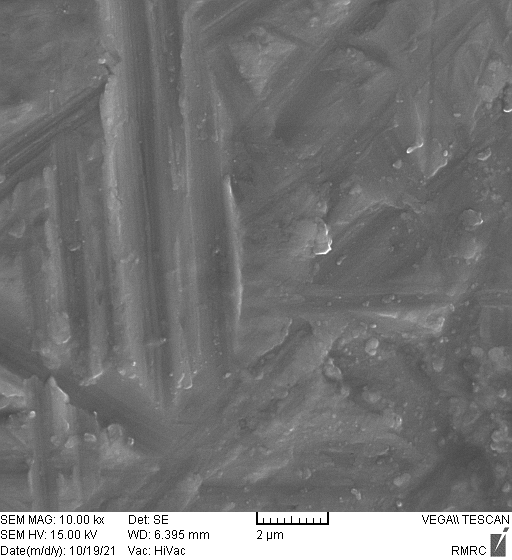

Supplement: Supplementary file 1 — Supplementary Material 1. [file 12903_2025_6614_MOESM1_ESM.tif]

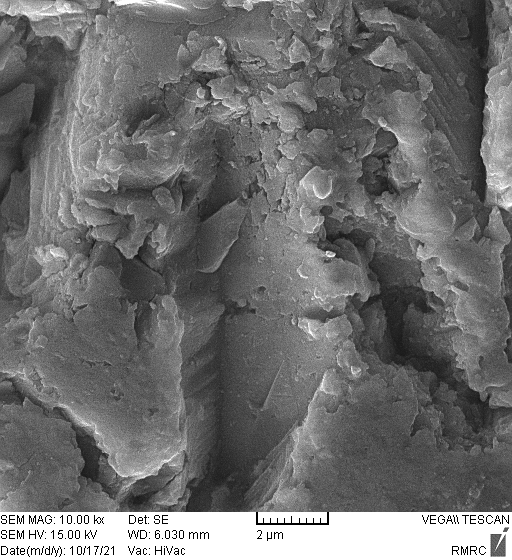

Supplement: Supplementary file 2 — Supplementary Material 2. [file 12903_2025_6614_MOESM2_ESM.tif]

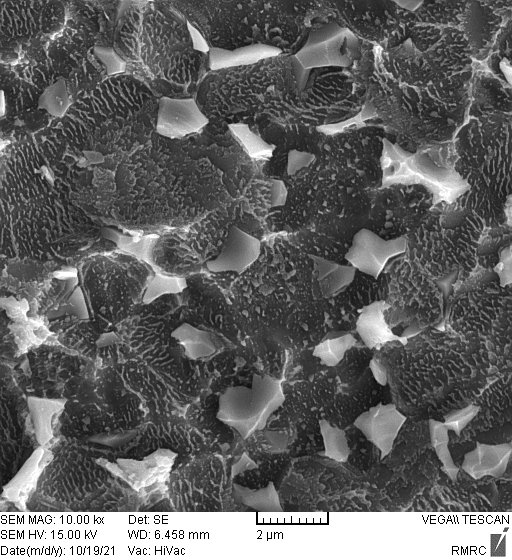

Supplement: Supplementary file 3 — Supplementary Material 3. [file 12903_2025_6614_MOESM3_ESM.tif]

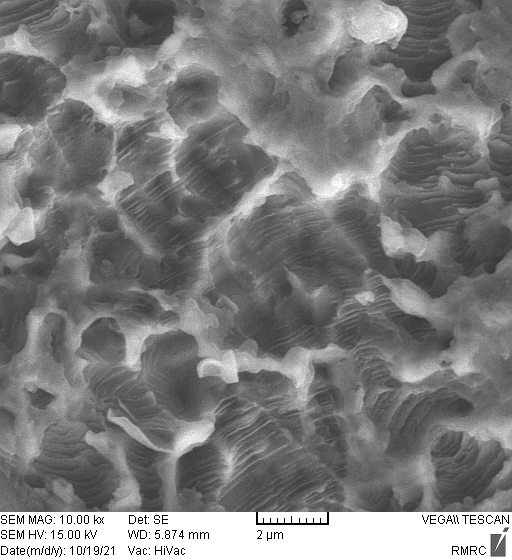

Supplement: Supplementary file 4 — Supplementary Material 4. [file 12903_2025_6614_MOESM4_ESM.tif]

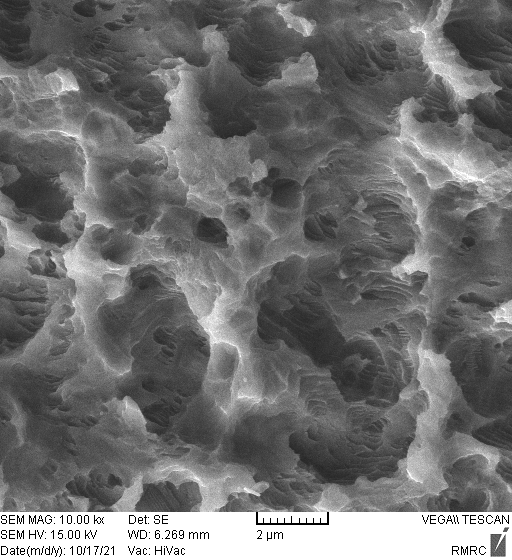

Supplement: Supplementary file 5 — Supplementary Material 5. [file 12903_2025_6614_MOESM5_ESM.tif]
